# Building Safe Emergency Medical Teams with Emergency Crisis Resource Management (E-CRM): An Interprofessional Simulation-Based Study

**DOI:** 10.3390/healthcare13151858

**Published:** 2025-07-30

**Authors:** Juan Manuel Cánovas-Pallarés, Giulio Fenzi, Pablo Fernández-Molina, Lucía López-Ferrándiz, Salvador Espinosa-Ramírez, Vanessa Arizo-Luque

**Affiliations:** 1 Faculty of Nursing, Catholic University of Murcia, Avenida de los Jeronimos, 135, 30107 Guadalupe, Spain; jmcanovas@ucam.edu (J.M.C.-P.); pfernandez@ucam.edu (P.F.-M.); llopez2@ucam.edu (L.L.-F.); varizo@ucam.edu (V.A.-L.); 2 Clinical Simulation Group of the Spanish Society of the Emergency Medicine (SEMES), Calle Luis de Salazar, 4, 28002 Madrid, Spain; 3 Emergency Medical Service of Madrid SUMMA112, Calle de la Antracita, 2 Bis, Arganzuela, 28045 Madrid, Spain

**Keywords:** simulation, interprofessional collaboration, interprofessional education, nursing, medicine, education

## Abstract

**Background/Objectives**: Effective teamwork is crucial for minimizing human error in healthcare settings. Medical teams, typically composed of physicians and nurses, supported by auxiliary professionals, achieve better outcomes when they possess strong collaborative competencies. High-quality teamwork is associated with fewer adverse events and complications and lower mortality rates. Based on this background, the objective of this study is to analyze the perception of non-technical skills and immediate learning outcomes in interprofessional simulation settings based on E-CRM items. **Methods**: A cross-sectional observational study was conducted involving participants from the official postgraduate Medicine and Nursing programs at the Catholic University of Murcia (UCAM) during the 2024–2025 academic year. Four interprofessional E-CRM simulation sessions were planned, involving randomly assigned groups with proportional representation of medical and nursing students. Teams worked consistently throughout the training and participated in clinical scenarios observed via video transmission by their peers. Post-scenario debriefings followed INACSL guidelines and employed the PEARLS method. **Results**: Findings indicate that 48.3% of participants had no difficulty identifying the team leader, while 51.7% reported minor difficulty. Role assignment posed moderate-to-high difficulty for 24.1% of respondents. Communication, situation awareness, and early help-seeking were generally managed with ease, though mobilizing resources remained a challenge for 27.5% of participants. **Conclusions**: This study supports the value of interprofessional education in developing essential competencies for handling urgent, emergency, and high-complexity clinical situations. Strengthening interdisciplinary collaboration contributes to safer, more effective patient care.

## 1. Introduction

Clinical simulation is an educational methodology that replicates or enhances real-world experiences within a controlled environment. From its origins with basic simulators to today’s high fidelity-technologically advanced systems, clinical simulation has undergone significant pedagogical and technological evolution [1,2]. Driven by the imperative of patient safety, clinical simulation enables the practice of both technical and non-technical skills in a risk-free setting [3]. Simulation Based Learning (SBL) has become one of the most important methodologies in the education and ongoing professional development of healthcare providers [4]. It encompasses a broad range of approaches, from low fidelity to complex virtual environments and high-fidelity team training in critical and complex scenarios [5].

In recent years, the demand for a more practical focus in health sciences education, coupled with technological advances, has further expanded the scope of clinical simulation. New methodologies such as Self-Learning Methodology in Simulated Environments (MAES©) and Virtual Reality Simulation (VR-S) have proven to be effective and well-accepted in both healthcare students and professionals’ training [6,7,8]. Among its many advantages, learning with simulation offers controlled exposure to rare critical situations and allows the repetition of procedures to ensure competency acquisition [9]. Regardless of the specific methodology employed, simulation fosters the development of essential skills (such as communication, leadership, and decision making) within a safe, structured environment [10]. Debriefing, one of the most important phases of clinical simulation, facilitates reflective learning helping knowledge retention [11].

To enhance team-based non-technical skills, clinical simulation can be integrated with the Crisis Resource Management (CRM) approach. Its origin is developed in the aviation industry [12] and later adapted to the healthcare setting. The CRM focuses on enhancing non-technical competencies such as effective communication, leadership, and teamwork. Those are vital in high-pressure, time-sensitive interprofessional settings [13]. To optimize human factors management in critical scenarios, the application of the CRM in medical training starts in anesthesiology [14] and subsequently expands to Emergency and Prehospital Care [15].

The implementation of the Crisis Resource Management (CRM) methodology, combined with clinical simulation, is essential and highly beneficial in emergency and critical care settings [16]. These environments are characterized by high-pressure and stressful situations, along with the imperative need for an effective multidisciplinary collaboration to enable rapid and accurate decision-making [17]. Traditional training models, which primarily emphasize technical skills, are insufficient to address the fact that approximately 70% of clinical errors are attributed to human factors, including deficits in communication, situational awareness, or leadership [18]. The CRM model specifically adapted to emergency and prehospital care is known as Emergency Crisis Resource Management (E-CRM). It aims to optimize the management of human factors in critical situations within emergency care environments [19]. The E-CRM, proposed by the Spanish Society of Emergency Medicine (SEMES), is structured around five validated strategic points: clear role definition, effective communication, appropriate task delegation, efficient use of available resources (including cognitive tools such as mobile applications and protocols), and situational awareness. By interacting with CRM methodology through E-CRM, an emergency medical team (EMT) can develop and refine critical non-technical skills. They enhance teamwork, which directly translates into a reduction in errors and a substantial improvement in patients’ safety and quality of care [20].

Given that an estimated 80–90% of errors in emergency care are attributed to human factors, the E-CRM model represents a valuable tool for mitigating risks and enhancing patient safety. Interprofessional education (IPE) applied in clinical simulation plays an important role in training for E-CRM. It enables the replication of realistic scenarios in a safe environment and allows for reflection about future job relationships. The CRM supports both skills practice and critical reflection through structured debriefing [15,21]. This concept has been extensively explored in the scientific literature, with numerous studies supporting the application of the CRM and simulation in the training of emergency teams [22]. Healthcare professionals, who trained in CRM principles, report significant improvements in their clinical performance, including increased use of support technologies and improved situational awareness and assessment [19,23].

Although direct evidence linking the CRM to a reduction in clinical errors and improved patient outcomes remains limited, existing research supports the premise that addressing human factors has an indirect yet significant impact on patient safety [24,25]. Given the significant role of human error in adverse events in critical or complex situations, and the recognized need to improve teamwork through effective leadership, communication, and task distribution, the following study was considered relevant. It examines the perception of non-technical skills and immediate learning outcomes in interprofessional clinical simulation scenarios, using the E-CRM framework. Specifically, this study explores how E-CRM-based simulations support the development of non-technical competencies. The analysis focuses on participants’ perceived skills acquisition and their reflections immediately following the simulation experience.

## 2. Materials and Methods

### 2.1. Study Design and Settings

This study employed a cross-sectional observational design following STROBE guidelines [26]. It involved students enrolled in the official Master’s Degree in Emergency Medicine and in the official Master’s Degree in Emergency Nursing in the Catholic University of Murcia (UCAM). The Official Master’s Degrees represent a formally recognized pathway for specialized training, endorsed by healthcare and academic institutions. Both Master’s Degrees are open to qualified professionals with clinical experience, allowing them to maintain their professional activities. Consequently, the content of these master’s programs has direct applicability to daily clinical practice. This study was conducted during the 2024–2025 academic year (from September 2024 to May 2025).

### 2.2. Participants

Participants were recruited voluntarily from official postgraduate Medicine and Nursing programs. For convenience sampling, two specific programs were selected: the Official Master’s Degree in Emergency Medicine and the Official Master’s Degree in Emergency Nursing. Two eligibility criteria were chosen. Firstly, students had to be enrolled in one of the two selected official programs at UCAM during the 2024–2025 academic year. Secondly, participants had to provide written informed consent. This consent explicitly emphasized the confidentiality requirements regarding the activities conducted during the study period. No specific exclusion criteria were applied.

### 2.3. Participants Allocation and Interprofessional Group Design

The authors include voluntary participants (medical and nursing students) and organized them into four subgroups including medical and nursing students in equal proportions in each one. Cluster sampling was used (medicine vs. nursing) followed by random selection of individuals within each cluster. As a result, the total of 29 participants was distributed into four subgroups (A, B, C, and D). There were three subgroups of 7 members each (4 medical and 3 nursing students) and one subgroup of 8 members (4 medical and 4 nursing students). Within each subgroup, participants were randomly assigned to four interprofessional teams as follows: two teams composed of two medical students and one nursing student (team “A1” and team “A2”), one team composed of one medical student and two nursing students (team “A3”), and one team composed of two medical students and two nursing students (team “A4”) (Table 1).

During the entire training period, the composition of teams in terms of professional roles (medical vs. nursing) remained fixed, although the individual members within each interprofessional team were allowed to vary based on the need of the simulation scenario.

This consistent grouping enabled the observation and analysis of participants’ behaviors and attitudes based on team composition and how interprofessional dynamics evolved depending on the presence of colleagues from the same or different disciplines.

### 2.4. Procedure

Four interprofessional, high-fidelity clinical simulation sessions were planned, following the E-CRM model. Each session lasted four hours and was co-facilitated by two certified clinical simulation instructors (one from the Medicine program and one from the Nursing program). Both instructors were accredited by the Spanish Society for Clinical Simulation and Patient Safety (SESSEP) and with more than 5 years-experience in clinical simulation. Each session consisted of four scenarios, lasting one hour each, resulting in a total of 16 simulation sessions (four per subgroup) and 64 individual simulation experiences.

Session 1 and 2 focused on development of non-technical skills using contexts that participants were already familiar with. These sessions followed team-based learning principles from the CRM model such as closed-loop communication, emotional support, situational leadership, and situational awareness. Session 3 and 4 introduced complex clinical scenarios that were more challenging for the participants, aligned with the academic content of the Emergency and Critical Care curriculum for both Medicine and Nursing. These included advanced life support, management of trauma patients, drowning, intentional poisoning, acute coronary syndrome, electrolyte imbalance, and non-invasive mechanical ventilation (Table 2).

All sessions were conducted in the clinical simulation facilities of UCAM (Murcia Campus), equipped with the necessary high-fidelity resources (actors, multiparametric monitors, real ambulances, etc.) to recreate realistic critical care scenarios. Simulation was designed in accordance with the International Nursing Association of Clinical Simulation and Learning (INACSL) standards [27]. It included structured phases of briefing (5 min) [28,29], scenario execution (15 min) [30], and debriefing (40 min) [31]. Each scenario adhered to specific learning objectives, although the order of presentation within each session was randomized. During each simulation one team participated actively, while the remaining teams observed in adjacent rooms via live video feed and patient monitoring systems. When simulations were conducted outdoors or in an ambulance, transmissions were streamed to designated observation rooms.

Debriefing was jointly conducted by both facilitators, using the Promoting Excellence and Reflective Learning in Simulation (PEARLS) framework [32,33]. The reaction phase was conducted jointly, allowing participants from both medicine and nursing to express their emotional response and initial impression of the scenario. The analysis phase followed a discipline-specific approach, enabling each professional group to reflect on the clinical situation from their own scopes of practice. Subsequently, the discussion process was integrated to promote interprofessional dialogue and comparison on the decision-making process. Finally, the summary phase was conducted collectively to consolidate shared learning objectives, identify cross-disciplinary takeaways, and foster a unified understanding of the case. INACSL guideline for effective debriefing and interprofessional education were followed at any time [31,34]. At the end of the four sessions, participants answered to a questionnaire.

### 2.5. Data Collection

A purpose-built ad hoc questionnaire was designed specifically for this study (questions included can be found in Supplementary Material). Clarity and usability were reviewed by members of the research team. Additionally, an external expert conducted a second review of the questionnaire to identify any errors or ambiguities. Following these two steps, the researchers revised the instrument to address any inconsistencies or unclear items. The principal objective of the questionnaire was to explore students’ perceptions, beliefs, and experiences regarding doctor–nurse interaction during interprofessional simulated clinical scenarios, based on the E-CRM validated items. The instrument consisted of 22 items divided into three main sections.

The first section gathers demographic information including age, sex, and academic program. The second section assessed stereotypes and preconceptions related to interprofessional collaboration in an E-CRM context. It included 15 statements rated on a five-point Likert scale (1 = Not at all difficult, 2 = Easy, 3 = Neutral, 4 = Difficult, and 5 = Very Difficult), aimed at measuring perceived challenges in teamwork between physicians and nurses. The third section included two open-ended questions designed to capture participants’ reflections on their simulation experience, including the emotions they experienced, the aspects they found most valuable, and suggestions for improving the interprofessional training, along with a rationale for their proposals.

The questionnaire was administered at the end of the interprofessional clinical simulation sessions. Participation was voluntary and anonymous, with prior informed consent integrated into the online form. It was distributed via web link sent by the research team to all participants, allowing only one response per institutional email address to ensure unique entries. From the date of distribution, participants had a 30-day period to respond. Any submission received after the deadline was excluded from the analysis to reduce recall bias and capture immediate post-simulation impressions. Data collection took place between 27 February and 31 March 2025.

This instrument allowed not only the qualification of students’ perceived difficulties and strengths related to clinical decision-making and team communication but also the identification of common emotional experiences and improvement proposals. These insights provided a more comprehensive understanding of the key factors shaping interprofessional interaction in high-fidelity simulated settings.

### 2.6. Data Analysis

The quantitative analysis was conducted using statistical correlation techniques [35] to evaluate the relationship among various variables related to performance in clinical simulations. Pearson correlation coefficients were applied to assess linear associations between experience, age, and performance. Spearman’s rank correlation coefficients were used to identify non-linear trends in technical and non-technical skills. The data were organized into correlation matrices and analyzed using statistical software tools. This allows a precise interpretation of the impact of training, workplace environment, and error management on the professional development of the participants. Given the limited sample size (n = 29), the risk of Type II error may increase. Non-significant findings were interpreted with caution, as they may not necessarily reflect the absence of an effect but rather insufficient power to detect it. The program IBM SPSS Statistics (v30) was used.

The qualitative data collected through the two open-ended questions in the final section of the questionnaire were analyzed using thematic content analysis. Participants’ responses were read to identify recurring themes and patterns. Codes were generated inductively, grouped into categories, and interpreted to capture the key insights expressed by the participants.

### 2.7. Ethical Considerations

Ethical approval for this study was granted by the Ethics Committee of the Universidad Católica de Murcia (UCAM) (CE: 122201). Participants who agreed to take part provided informed consent to receive the web-based questionnaire link via email. Prior to completing the questionnaire, a statement was presented outlining the purpose of this study, assurances of confidentiality, the voluntary nature of participation, and clarification that non-submission would carry no consequences. Acceptance of these conditions was considered equivalent to informed consent for participation in this study. Participants could withdraw from this study at any time.

## 3. Results

### 3.1. General

The analysis of the questionnaires revealed various correlations between demographic variables, CRM components, and clinical performance (Table 3).

Furthermore, it was possible to establish correlations between the impact of training and the participants’ professional performance in healthcare settings (Table 4).

### 3.2. Demographic Information

A total of 29 participants were included, representing approximately 72% of the total student population enrolled in these programs (9 from the Master’s in Emergency Medicine and 20 from the Master’s in Emergency Nursing). The sample was predominantly female: 75.9% women (n = 22) and 24.1% men (n = 7). Most respondents were between 22 and 25 years old (41.4%, n = 12), followed by those over 36 years (31%, n = 9), those aged 26–30 (20.7%, n = 6), and a minority aged 31–35 (6.9%, n = 2).

Most participants were enrolled in a nursing master’s degree (69%, n = 20), while 31% (n = 9) were pursuing a master’s degree in medicine. All participants were actively working within the healthcare system: 27.6% in hospital emergency departments, 34.5% in prehospital emergency services, 34.5% in other healthcare services, and 3.4% in intensive care units.

Emergency care experience was predominantly low, with 89.7% (n = 26) of participants having between 0 and 5 years of experience. Only 6.9% had 5 to 10 years of experience, and 3.4% had 10 to 15 years. None had more than 15 years of experience. These data indicate that the participants were professionals in the early stages of their careers in emergency care. Although participants had less than 15 years of experience in emergency care, this study revealed a significant negative correlation between professional experience and perceived difficulties in scenario performance (r = −0.72, *p* < 0.01), indicating that professionals with more years of practice reported fewer challenges related to both technical and non-technical skills.

### 3.3. Interprofessional Collaboration in E-CRM

Professionals working in hospital and prehospital emergency services reported a higher perception of scenarios’ difficulty compared to those in other clinical areas (r = −0.41, *p* < 0.05). A moderate positive correlation was observed between accurate identification of clinical severity and the development of appropriate response (r = 0.62, *p* < 0.01), suggesting that effective clinical judgement enhances intervention quality

Following the simulations, 48.3% (n = 14) of participants found **identifying the team leader** “not at all difficult”, and 51.7% (n = 15) rated it as “easy”. These results indicates that leadership recognition was generally manageable. Notably, a significant correlation (r = −0.67, *p* < 0.05) suggested that increased professional experience was associated with greater ease in identifying leadership roles, while less experienced professionals encountered more difficulty.

Regarding the explicit **assignment of roles**, 27.6% (n = 8) reported “not at all difficult”, 48.3% (n = 14) “easy”, 20.7% (n = 6) “normal”, and 3.4% (n = 1) experienced it as difficult. While most participants were able to assign roles, a significant proportion experienced some level of complexity in performing this task.

In terms of **task distribution**, difficulty perception was low: 31% (n = 9) reported no difficulty, 55.2% (n = 16) “easy”, and 13.8% (n = 4) “normal”. Similarly, cross-checking actions were broadly perceived as manageable, with 17.2% (n = 5) selecting “no difficulty at all”, 58.6% (n = 17) “easy”, 20.7% (n = 6) “normal”, and 3.4% (n = 1) “difficult”. A positive correlation between clear task distribution and the development of effective cross-checks (r = 0.55, *p* < 0.05) highlighted the relevance of structured team coordination in minimizing procedural errors and promoting patient safety.

**Effective communication** via the designated leader was considered “not at all difficult” by 37.9% (n = 11) and “easy” by 44.8% (n = 13); 17.2% (n = 5) reported difficulty. A similar pattern emerged in relation to early help-seeking with 34.5% (n = 10) indicating “not at all difficult”, 44.8% (n = 13) “easy”, 17.2% (n = 5) “normal”, and 3.4% (n = 1) “difficult”. These findings reinforce the earlier correlation between organized task distribution and effective procedural execution (r = 0.55, *p* < 0.05).

Regarding **situational awareness**, 37.9% (n = 11) had no difficulty identifying the clinical environment, 44.8% (n = 13) rated it as “easy”, and 17.2% (n = 5) “normal”. Mobilizing necessary resources proved more challenging, with only 24.1% (n = 7) reporting no difficulty, 48.3% (n = 14) easy, 24.1% (n = 7) normal, and 3.4% (n = 1) difficult. Again, a strong positive correlation was found between correct assessment of patient severity and effective clinical management (r = 0.62, *p* < 0.01). Additionally, the ability to manage errors was positively associated with prioritization in clinical decision-making (r = 0.68, *p* < 0.01).

**Anticipation and planning** emerged as one of the most challenging aspects, with only 17.2% (n = 5) reporting no difficulty, 37.9% (n = 11) easy, 34.5% (n = 10) normal, and 10.3% (n = 3) experiencing difficulties. A similar trend was observed for error prevention and correction, with 24.1% (n = 7) reporting “not at all difficult”, 34.5% (n = 10) easy, 37.9% (n = 11) normal, and 3.4% (n = 1) difficult.

**Task handovers** were reported as “not at all difficult” by 20.7% (n = 6), “easy” by 51.7% (n = 15), “normal” by 24.1% (n = 7), and 3.4% (n = 1) difficult. The ability to maintain task focus was generally adequate, with 31% (n = 9) reporting no difficulty, 48.3% (n = 14) easy, 17.2% (n = 5) normal, and 3.4% (n = 1) difficult.

**Dynamic prioritization** was mostly unproblematic, with 34.5% (n = 10) of participants rating it as “not at all difficult”, 44.8% (n = 13) “easy”, and 20.7% (n = 6) “normal”.

Analysis revealed several significant correlations emphasizing the importance of integrated training in emergency care. The ability to accurately assess patient severity was strongly associated with effective management strategies (r = 0.62, *p* < 0.01), while error correction correlated positively with prioritized clinical decision-making (r = 0.68, *p* < 0.01). Complementary training, particularly in emergency management, leadership, and advanced simulation, was linked to improved team coordination *(r* = 0.61, *p* < 0.01), leader identification *(r* = 0.58, *p* < 0.01), and reduced decision-making errors *(r* = 0.66, *p* < 0.01). Additionally, emergency training was associated with the development of dynamic responses under pressure *(r* = 0.63, *p* < 0.01). A positive correlation between technical and non-technical difficulties *(r* = 0.58, *p* < 0.01) further suggests that clinical performance issues often coincide with challenges in communication, leadership, and teamwork, highlighting the need for comprehensive training programs that address both skill domains.

Notably, no participants selected the “very difficult” option on any item, indicating that while variability in perceived difficulty existed, overall responses remained within the manageable range.

### 3.4. Participants Perceptions

Participants highlighted several positive aspects of the simulation experience, emphasizing improvements in teamwork and personal growth, particularly in overcoming fear related to professional challenges. They reported enhanced skills in organization, leadership, safety, and clinical decision-making. The simulation also fostered greater confidence in interprofessional communication, especially between nurses and physicians, reducing hierarchical barriers and encouraging open idea-sharing.

Some participants suggested improvements, including increasing session frequency and duration, to cover more scenarios without compromising focus. Others stressed the importance of active listening, particularly among medical students toward nursing ones.

## 4. Discussion

Clinical simulation is a fundamental tool for enhancing patient safety and the quality of care, as it enables healthcare professionals to train both technical and non-technical skills in a controlled, risk-free environment. This practice facilitates the identification of potential errors before they occur in real clinical settings, thereby promoting that simulation reduces adverse events and improves clinical outcomes. It is established as a key component in the training and assessment of clinical performance [36,37].

Crisis Resource Management (CRM) training has proven to be a valuable tool in improving clinical performance, particularly in high-pressure and emergency situations. Integrating CRM principles into simulation-based education allows healthcare professionals to develop essential non-technical skills such as communication, leadership, decision-making, and teamwork. These competencies are critical for ensuring patient safety and effective crisis management, underscoring the importance of incorporating CRM training into healthcare curricula through realistic and structured simulation scenarios [38,39].

The findings of this study reaffirm the pedagogical value of advanced clinical simulation as a fundamental tool in the education of healthcare professionals, particularly in emergency and critical care settings. The sample, composed primarily of postgraduate nursing students, revealed a clear association between professional experience, leadership perception, role identification, and performance within simulated scenarios.

The negative correlation between professional experience and perceived difficulties (r = −0.72, *p* < 0.01) is consistent with the recent literature. It emphasizes that accumulated clinical experience enhances the integration of both technical and non-technical skills in highly stressful situations [30,40]. This has direct implications for the improvement of core competencies such as dynamic prioritization, recognition of clinical severity, and decision-making under pressure.

Additionally, a clear distribution of tasks within the team was positively correlated with the development of cross-checks (r = 0.55, *p* < 0.05), aligning with current collaborative models in emergency medicine [41]. Cross-checks are essential in preventing medical errors, and their effectiveness is enhanced by proper communication, coordination, and leadership, that are strengthened through advanced simulation training.

One of the most notable results is the positive correlation between training in advanced simulation and the reduction in decision-making errors (r = 0.66, *p* < 0.01), reinforcing the evidence that deliberate practice in controlled environments improves clinical responsiveness [42]. Through simulated experiences, learners internalize not only clinical routines but also key organizational behaviors such as time management, leadership, and interprofessional communication.

Within the E-CRM framework, the integration of digital platforms for performance tracking and personalized feedback represents a significant educational innovation. Although the present study did not directly assess the impact of such platforms; it can be inferred that the use of technologies may enhance adaptive learning by tailoring simulations to individual user needs [43]. Recent studies advocate for the use of CRM to improve and monitor of both clinical and non-clinical competencies [44,45].

It was also found that the ability to anticipate and plan correlates with the previous experience in simulation and specialized emergency training. This finding supports the need to incorporate more advanced simulation sessions into postgraduate academic programs, increasing exposure to complex scenarios that promote strategic planning in high-pressure context [46,47].

From a training perspective, the integration of technical (clinical procedures) and non-technical (leadership, communication, and stress management) skills must be prioritized. The study data reveal a positive correlation between difficulties in technical and non-technical skills (r = 0.58), supporting the theory that these dimensions are interdependent [48]. Therefore, clinical simulation training should not be approached in a fragmented manner but rather through a systematic and interdisciplinary framework [49].

Finally, the analysis of open-ended response reflects a generally positive perception of the simulation experience, particularly in terms of self-confidence, team integration, and personal growth. These perceived benefits are consistent with documented effects of experiential learning in simulated environment [50].

### Limitation

This study has limitations that warrant consideration. Although the questionnaire included items previously validated in the E-CRM instrument, the remaining variables were limited to sociodemographic and qualitative data, which do not require formal validation. Clarity and usability were ensured through internal review and external expert evaluation; however, the use of a partially adapted tool may still limit the overall reliability of the data.

Additionally, this study focused only on immediate learning outcomes, without assessing long-term retention or the transfer of acquired competencies to clinical practice. The modest sample size, predominantly composed of postgraduate nursing students, further limits the generalizability of the findings and increases the risk of Type II error. Despite these constraints, the participants’ professional background in emergency care offers contextually relevant insights. Future research should include longitudinal designs and more diverse interprofessional samples to validate and expand upon these findings.

## 5. Conclusions

This study provides preliminary insights into the pedagogical relevance and potential clinical value of advanced simulation-based training incorporating E-CRM, particularly in the development of both technical and non-technical competencies among postgraduate students in emergency care contexts. Meanwhile, the findings suggest possible associations between prior clinical experience, leadership identification, and role distribution with improved performance in simulated scenarios. These observations should be interpreted within the context of a modest sample size and specific study population. The questionnaire, which was purpose-built, did not follow a formal validation procedure.

Interpofessional simulation training using the E-CRM framework appeared to support improvements in decision-making process, team coordination, communication, and task delegation. The structured integration of advanced simulation, debriefing, and E-CRM may offer a useful approach to support learning and skills development in high-pressure environments. Furthermore, the interplay observed between technical and non-technical skills suggests the potential relevance of interprofessional educational strategies.

Simulation-based interprofessional education (IPE), particularly when combined with E-CRM, may contribute to enhance learning of non-technical competences, patient’s safety, and collaborative clinical decision-making. However, these conclusions remain context-specific and should not be generalized beyond the scope of this study. Future research is needed to explore the integration of adaptative technologies in competency-based training and to further investigate the applicability of E-CRM in undergraduate emergency simulation, using both qualitative and quantitative approaches.

## Figures and Tables

**Table 1:** Group division.

Groups	Components	Teams/Participants
A	4 Doctors and 3 Nurses	Teams A1, B1, C1, D1: 2 Doctors and 1 Nurse
B	4 Doctors and 3 Nurses	Teams A2, B2, C2, D2: 2 Doctors and 1 Nurse
C	4 Doctors and 3 Nurses	Teams A3, B3, C3, D3: 1 Doctors and 2 Nurse
D	4 Doctors and 4 Nurses	Teams A4, B4, C4, D4: 2 Doctors and 2 Nurse

**Table 2:** Simulation scenarios.

Session 1	Session 2	Session 3	Session 4
Hypoglicemia Metabolic Ketoacidosis Ischemic Stroke Drug Intoxication	Bradyarrhythmia Hemorrhagic Stroke Tachyarrhythmia	Acute Pulmonary Edema Life-Threatening Asthma Attack Severe Burn	Chest Trauma Abdominal Trauma Severe Traumatic Brain Injury

**Table 3:** Correlation analysis of variables.

Variable 1	Variable 2	Correlation Coefficient (r)	*p*-Value [CI* 95%]	Interpretation
Professional experience	Performance	−0.72	0.0003 [−0.86, −0.48]	Greater experience is associated with fewer perceived difficulties.
Professional experience	Leader identification	−0.67	0.0011 [−0.83, −0.39]	More experience facilitates the recognition of leadership roles.
Professional experience	Perceived difficulties	−0.68	0.0009 [−0.84, −0.41]	Increased experience correlates with fewer reported difficulties.
Age	Performance	0.65	0.0018 [0.35, 0.82]	Older participants report higher perceived performance.
Degree type (Medicine/Nursing)	Performance	−0.27	0.1427 [−0.61, 0.10]	No significant relationship between degree type and performance.
Work area	Performance	−0.41	0.0329 [−0.70, −0.04]	High-pressure environments increase the perception of difficulty.
Type of master’s program	Communication skills	−0.32	0.0875 [−0.65, 0.04]	No strong correlation between master’s program type and communication skills.
Type of master’s program	Leadership	−0.38	0.0493 [−0.69, −0.003]	The type of master’s program does not significantly influence leadership skills.
Work distribution	Development of cross-checks	0.55	0.0067 [0.18, 0.78]	Effective work distribution enhances process validation through cross-checking.
Severity identification	Severity management	0.62	0.0022 [0.28, 0.82]	Accurate identification of severity improves clinical management.
Error correction	Dynamic prioritization in scenarios	0.68	0.0008 [0.39, 0.85]	Error correction enhances prioritization in critical situations.
Difficulties in technical skills	Difficulties in non-technical skills	0.58	0.0043 [0.23, 0.79]	Participants with technical difficulties also face challenges in communication and leadership.

* Confidence interval 95%.

**Table 4:** Effect of supplementary training on professional performance.

Variable 1	Variable 2	Correlation Coefficient (r)—*p*-Value [CI* 95%]	Interpretation
Training in emergency management	Team coordination	0.61—*p* = 0.0029 [0.27, 0.81]	Greater emergency training is associated with improved task distribution.
Leadership training	Identification of the leader in critical scenarios	0.58—*p* = 0.0046 [0.24, 0.79]	Leadership training enhances the ability to recognize leadership roles.
Training in advanced simulations	Reduction in decision-making errors	0.66—*p* = 0.0012 [0.34, 0.84]	Simulation experience improves error correction and prioritization capabilities.
Emergency management training	Development of dynamic responses in clinical settings	0.63—*p* = 0.0021 [0.30, 0.82]	Emergency training facilitates adaptability in high-pressure situations.

* Confidence interval 95%.

## Data Availability

Data is contained within the article or Supplementary Material.

## Supplementary Materials

The following supporting information can be downloaded at: https://www.mdpi.com/article/10.3390/healthcare13151858/s1, E-CRM INTERPROFESSIONAL CLINICAL SIMULATION QUESTIONNAIRE.

## Abbreviations

The following abbreviations are used in this manuscript:

CRM	Crisis Resource Management
E-CRM	Emergency Crisis Resource Management
EMT	Emergency Medical Team
INACSL	The International Nursing Association of Clinical Simulation and Learning
IPE	Interprofessional Education
MAES©	Self-Learning Methodology in Simulated Environments
PEARLS	Promoting Excellence and Reflective Learning in Simulation
SBL	Simulation Based Learning
SEMES	Spanish Society of Emergency Medicine
SESSEP	Spanish Society for Clinical Simulation and Patient Safety
UCAM	Catholic University of Murcia
VR-S	Virtual Reality Simulation

## References

[1] Cooper J.B., Taqueti V.R. (2008). A Brief History of the Development of Mannequin Simulators for Clinical Education and Training. Postgrad. Med. J..

[2] Bienstock J., Heuer A. (2022). A Review on the Evolution of Simulation-Based Training to Help Build a Safer Future. Medicine.

[3] Saleem M., Khan Z. (2023). Healthcare Simulation: An Effective Way of Learning in Health Care. Pak. J. Med. Sci..

[4] Alshehri F.D., Jones S., Harrison D. (2023). The Effectiveness of High-Fidelity Simulation on Undergraduate Nursing Students’ Clinical Reasoning-Related Skills: A Systematic Review. Nurse Educ. Today.

[5] Haerkens M.H.T.M., Kox M., Lemson J., Houterman S., van der Hoeven J.G., Pickkers P. (2015). Crew Resource Management in the Intensive Care Unit: A Prospective 3-Year Cohort Study. Acta Anaesthesiol. Scand..

[6] Sharoff L. (2022). Student’s Perception of vSim for Nursing^®^ Using the Simulation Effectiveness Tool—Modified. Clin. Simul. Nurs..

[7] Díaz J.L., Leal C., García J.A., Hernández E., Adánez M.G., Sáez A. (2016). Self-Learning Methodology in Simulated Environments (MAES©): Elements and Characteristics. Clin. Simul. Nurs..

[8] Fenzi G., Reuben A.D., Agea J.L.D., Ruipérez T.H., Costa C.L. (2022). Self-Learning Methodology in Simulated Environments (MAES©) Utilized in Hospital Settings. Action-Research in an Emergency Department in the United Kingdom. Int. Emerg. Nurs..

[9] Zeng Q., Wang K., Liu W., Zeng J., Li X., Zhang Q., Ren S., Xu W. (2023). Efficacy of High-Fidelity Simulation in Advanced Life Support Training: A Systematic Review and Meta-Analysis of Randomized Controlled Trials. BMC Med. Educ..

[10] Stefanidis D., Cook D., Kalantar-Motamedi S.-M., Muret-Wagstaff S., Calhoun A.W., Lauridsen K.G., Paige J.T., Lockey A., Donoghue A., Hall A.K. (2024). Society for Simulation in Healthcare Guidelines for Simulation Training. Simul. Healthc..

[11] Fegran L., ten Ham-Baloyi W., Fossum M., Hovland O.J., Naidoo J.R., van Rooyen D.R.M., Sejersted E., Robstad N. (2023). Simulation Debriefing as Part of Simulation for Clinical Teaching and Learning in Nursing Education: A Scoping Review. Nurs. Open.

[12] Kanki B.G., Anca J., Helmreich R.L. (2010). Crew Resource Management.

[13] Bochatay N., Ju M., O’Brien B.C., van Schaik S.M. (2025). A Scoping Review of Interprofessional Simulation-Based Team Training Programs. Simul. Healthc..

[14] Gaba D.M., Fish K.J., Howard S.K., Burden A. (2014). Crisis Management in Anesthesiology E-Book: Crisis Management in Anesthesiology E-Book.

[15] Casal Angulo C., Quintillá Martínez J.M., Espinosa Ramírez S. (2020). Clinical Simulations and Safety in Emergencies: Emergency Crisis Resource Management. Emergencias.

[16] Watkins S.C., Hensley N.B. (2023). Team Dynamics in the Operating Room: How Is Team Performance Optimized?. Anesthesiol. Clin..

[17] Truta T.S., Boeriu C.M., Lazarovici M., Ban I., Petrişor M., Copotoiu S.-M. (2018). Improving Clinical Performance of an Interprofessional Emergency Medical Team Through a One-Day Crisis Resource Management Training. J. Crit. Care Med. Univ. Med. Si Farm. Din Targu-Mures.

[18] Lei C., Palm K. (2025). Crisis Resource Management Training in Medical Simulation. StatPearls.

[19] Espinosa Ramírez S., Casal Angulo M.d.C., Díaz Agea J.L., Vázquez Casares A.M., López Mesa F., Adánez Martínez M.G. (2023). E-CRM como herramienta de cambio actitudinal en los equipos de urgencia. Rev. Esp. Urgenc. Emerg..

[20] Amaro-López L., Hernández-González P.L., Hernández-Blas A., Arzola L.I.H. (2019). La simulación clínica en la adquisición de conocimientos en estudiantes de la Licenciatura de Enfermería. Enferm. Univ..

[21] Fenzi G., Díaz-Agea J.L., Pethick D., Bertolín-Delgado R., Hernández-Donoso N., Lorente-Corral L. (2022). An Undergraduate Interprofessional Experience with Self-Learning Methodology in Simulation Environment (MAES©): A Qualitative Study. Nurs. Rep. Pavia Italy.

[22] Jankouskas T., Bush M.C., Murray B., Rudy S., Henry J., Dyer A.M., Liu W., Sinz E. (2007). Crisis Resource Management: Evaluating Outcomes of a Multidisciplinary Team. Simul. Healthc..

[23] Carne B., Kennedy M., Gray T. (2012). Review Article: Crisis Resource Management in Emergency Medicine. Emerg. Med. Australas..

[24] Gross B., Rusin L., Kiesewetter J., Zottmann J.M., Fischer M.R., Prückner S., Zech A. (2019). Crew Resource Management Training in Healthcare: A Systematic Review of Intervention Design, Training Conditions and Evaluation. BMJ Open.

[25] Fung L., Boet S., Bould M.D., Qosa H., Perrier L., Tricco A., Tavares W., Reeves S. (2015). Impact of Crisis Resource Management Simulation-Based Training for Interprofessional and Interdisciplinary Teams: A Systematic Review. J. Interprof. Care.

[26] von Elm E., Altman D.G., Egger M., Pocock S.J., Gøtzsche P.C., Vandenbroucke J.P., STROBE Initiative (2007). The Strengthening the Reporting of Observational Studies in Epidemiology (STROBE) Statement: Guidelines for Reporting Observational Studies. Ann. Intern. Med..

[27] Healthcare Simulation Standards of Best PracticeTM-Clinical Simulation in Nursing. https://www.nursingsimulation.org/article/S1876-1399(21)00105-5/fulltext.

[28] McDermott D.S., Ludlow J., Horsley E., Meakim C. (2021). Healthcare Simulation Standards of Best Practice^TM^ Prebriefing: Preparation and Briefing. Clin. Simul. Nurs..

[29] Rutherford-Hemming T., Lioce L., Breymier T. (2019). Guidelines and Essential Elements for Prebriefing. Simul. Healthc. J. Soc. Simul. Healthc..

[30] Watts P.I., McDermott D.S., Alinier G., Charnetski M., Ludlow J., Horsley E., Meakim C., Nawathe P.A. (2021). Healthcare Simulation Standards of Best Practice^TM^ Simulation Design. Clin. Simul. Nurs..

[31] Decker S., Alinier G., Crawford S.B., Gordon R.M., Jenkins D., Wilson C. (2021). Healthcare Simulation Standards of Best Practice^TM^ The Debriefing Process. Clin. Simul. Nurs..

[32] Cheng A., Grant V., Robinson T., Catena H., Lachapelle K., Kim J., Adler M., Eppich W. (2016). The Promoting Excellence and Reflective Learning in Simulation (PEARLS) Approach to Health Care Debriefing: A Faculty Development Guide. Clin. Simul. Nurs..

[33] Eppich W., Cheng A. (2015). Promoting Excellence and Reflective Learning in Simulation (PEARLS): Development and Rationale for a Blended Approach to Health Care Simulation Debriefing. Simul. Healthc. J. Soc. Simul. Healthc..

[34] Rossler K., Molloy M.A., Pastva A.M., Brown M., Xavier N. (2021). Healthcare Simulation Standards of Best Practice^TM^ Simulation-Enhanced Interprofessional Education. Clin. Simul. Nurs..

[35] Miot H.A. (2018). Correlation Analysis in Clinical and Experimental Studies. J. Vasc. Bras..

[36] Dalwood N., Bowles K.-A., Williams C., Morgan P., Pritchard S., Blackstock F. (2020). Students as Patients: A Systematic Review of Peer Simulation in Health Care Professional Education. Med. Educ..

[37] Meling T.R., Meling T.R. (2021). The Impact of Surgical Simulation on Patient Outcomes: A Systematic Review and Meta-Analysis. Neurosurg. Rev..

[38] Verbeek-van Noord I., de Bruijne M.C., Zwijnenberg N.C., Jansma E.P., van Dyck C., Wagner C. (2014). Does Classroom-Based Crew Resource Management Training Improve Patient Safety Culture? A Systematic Review. SAGE Open Med..

[39] Abildgren L., Lebahn-Hadidi M., Mogensen C.B., Toft P., Nielsen A.B., Frandsen T.F., Steffensen S.V., Hounsgaard L. (2022). The Effectiveness of Improving Healthcare Teams’ Human Factor Skills Using Simulation-Based Training: A Systematic Review. Adv. Simul..

[40] Leonardsen A.-C.L. (2023). The Impact of Clinical Experience in Advanced Practice Nursing Education-A Cross-Sectional Study of Norwegian Advanced Practice Nurses’ Perspectives. Nurs. Rep. Pavia Italy.

[41] Freund Y., Rousseau A., Berard L., Goulet H., Ray P., Bloom B., Simon T., Riou B. (2015). Cross-Checking to Reduce Adverse Events Resulting from Medical Errors in the Emergency Department: Study Protocol of the CHARMED Cluster Randomized Study. BMC Emerg. Med..

[42] Zhao W., Xu M., Tian Q., Han Y., Wang Z., Zhang W. (2024). The Impact of Simulation-Based Learning on Nursing Decision-Making Ability: A Meta-Analysis. Clin. Simul. Nurs..

[43] Halkiopoulos C., Gkintoni E. (2024). Leveraging AI in E-Learning: Personalized Learning and Adaptive Assessment through Cognitive Neuropsychology—A Systematic Analysis. Electronics.

[44] Huffman E.M., Anton N.E., Athanasiadis D.I., Ahmed R., Cooper D., Stefanidis D., Lee N.K. (2021). Multidisciplinary Simulation-Based Trauma Team Training with an Emphasis on Crisis Resource Management Improves Residents’ Non-Technical Skills. Surgery.

[45] Sánchez-Marco M., Escribano S., Rubio-Aparicio M., Juliá-Sanchis R., Cabañero-Martínez M.-J. (2023). Effectiveness of Nontechnical Skills Educational Interventions in the Context of Emergencies: A Systematic Review and Meta-Analysis. Aust. Crit. Care.

[46] Elendu C., Amaechi D.C., Okatta A.U., Amaechi E.C., Elendu T.C., Ezeh C.P., Elendu I.D. (2024). The Impact of Simulation-Based Training in Medical Education: A Review. Medicine.

[47] Diaz-Navarro C., Jones B., Pugh G., Moneypenny M., Lazarovici M., Grant D.J. (2024). Improving Quality through Simulation; Developing Guidance to Design Simulation Interventions Following Key Events in Healthcare. Adv. Simul..

[48] Gamborg M.L., Salling L.B., Rölfing J.D., Jensen R.D. (2024). Training Technical or Non-Technical Skills: An Arbitrary Distinction? A Scoping Review. BMC Med. Educ..

[49] Saragih I.D., Suarilah I., Hsiao C.-T., Fann W.-C., Lee B.-O. (2024). Interdisciplinary Simulation-Based Teaching and Learning for Healthcare Professionals: A Systematic Review and Meta-Analysis of Randomized Controlled Trials. Nurse Educ. Pract..

[50] Zafošnik U., Cerovečki V., Stojnić N., Belec A.P., Klemenc-Ketiš Z. (2024). Developing a Competency Framework for Training with Simulations in Healthcare: A Qualitative Study. BMC Med. Educ..

