# Supplementary material for: Building Safe Emergency Medical Teams with Emergency Crisis Resource Management (E-CRM): An Interprofessional Simulation-Based Study
Source: Healthcare (Basel). 2025 Jul 30;13(15):1858. doi: 10.3390/healthcare13151858 (PMC12346667; doi:10.3390/healthcare13151858)
Supplement: Supplementary file 1 [file healthcare-13-01858-s001.zip › healthcare-3720906-supplementary.pdf]

E-CRM INTERPROFESSIONAL CLINICAL SIMULATION QUESTIONNAIRE

Section 1

1. Age
    a. 22-25
    b. 26-30
    c. 31-35
    d. >36
2. Gender
    a. Masculine
    b. Feminine
3. What are you studying?
    a. Emergency Medicine Master
    b. Emergency Nursing Master
4. Where are you working?
    a. Accident & Emergency
    b. Ambulance Services
    c. Intensive Care Unite
    d. Others
5. Emergency Experience
    a. Less than 5 years
    b. 5 to less than 10 years
    c. 10 to less than 15 years
    d. 15 years or more

Section 2

Answer the following questions marking one of the Linket Scale indicators:

- 1= Not at all difficult
- 2= Easy
- 3= Neutral
- 4= Difficult
- 5= Very Difficult

Questions:

1. Identify the leader
2. Explicit assignment of roles
3. I*dentification of workload distribution*
4. Identification and implementation of cross-checks
5. Effective communication through the team leader
6. Early recognition of severity and timely request for help
7. Identification of the environment
8. Mobilization of available and necessary resources
9. Use of cognitive aids during the scenario
10. Ability to use all available information
11. Development of anticipation and planning during the scenario
12. Development of error prevention, correction, and fixation strategies

13. Execution of continuous task handovers
14. Sustained focus on the tasks being performed
15. Dynamic prioritization during the scenario

Section 3

Open ended questions

1. What positive aspects would you highlight from this joint simulation experience?
2. What aspects do you think could be improved? Why?
